# Assessing Temperament Risk Factors in Late Childhood and Early Adolescence: Development and Validation of the Integrative Late Childhood Temperament Inventory

**DOI:** 10.1007/s10578-024-01675-5

**Published:** 2024-04-15

**Authors:** Vivienne Biedermann, Marcel Zentner

**Affiliations:** Department of Psychology, University of Innsbruck, Universitätsstrasse 15, 6020 Innsbruck, Austria

**Keywords:** Child temperament, Behavior problems, Emotional problems, School failure, Assessment, Child development

## Abstract

Childhood temperament has a well-documented influence on later life outcomes, notably behavior problems, quality of interpersonal relationships, and academic achievement. However, there is at present a lack of brief measures that assess temperament characteristics which are represented across models of temperament in the late childhood and early adolescence period. To redress this gap, the aim of the current study was to develop and validate a short questionnaire that extends an earlier integrative measure of temperament, the Integrative Child Temperament Inventory (ICTI) to the late childhood period. The new measure (named Integrative Late Childhood Temperament Inventory, ILCTI) includes all five components of the ICTI (frustration, behavioral inhibition, attention/persistence, activity level, sensory sensitivity), plus affiliation. German- or English-speaking parents rated a total of 525 children, aged 8 to 14 years. Confirmatory factor analyses supported the predicted structure of the new measure. Examination of fit indices and psychometric analyses showed that 24 items are sufficient to capture all six dimensions reliably. Next to convergent validity with established measures of temperament and personality, we also present analyses of associations with measures of externalizing problems, internalizing problems, and school failure all of which support the instrument’s clinical utility. Thus, despite its brevity, the ILCTI is a reliable and valid measure of late childhood temperament that can be helpful in assessing risk of developing behavior problems or school failure.

**Supplementary Information:**

The online version contains supplementary material available at 10.1007/s10578-024-01675-5.

## Introduction

The temperament of a child has a substantial influence on outcomes later in life, such as adult personality, behavior problems, scholastic and occupational achievement, health, and relationships [1]. For example, high frustration and fear/inhibition, as well as low effortful control in late childhood and early adolescence are related to internalizing problems, while high frustration and low effortful control are related to externalizing problems [2]. More specifically, anger and/or frustration in childhood has been shown to predict antisocial behaviors, behavioral inhibition has been found to foreshadow anxiety disorders, low positive emotionality increases the risk for depression, whereas activity level is predictive of attention-deficit/hyperactivity disorder (ADHD) and antisocial behavior. Effortful control reduces the risk for all of these disorders [3]. Within a class of disorders, temperament has also been found to be predictive of differential symptomatology. For example, ADHD inattention symptoms in school-aged children are more specifically related to low effortful control, whereas ADHD hyperactive-impulsive symptoms show a greater association with lack of inhibition [4]. A difficult temperament in toddlers (i.e., a combination of high reactivity and low attention, low adaptability, and low inhibition) is related to autism spectrum disorder (ASD) with disruptive behavior in school-aged children, but not to ASD without disruptive behavior [5]. In response to these findings, various temperament-based interventions were introduced in recent years, from parent- and teacher-guidance [6], behavioral skills training [7], to computer exercises aiming to foster self-regulation (e.g., [8]) or decrease behavioral inhibition (e.g., [9]).

An effective integration of child temperament into clinical research, assessments, and treatment approaches is contingent on the availability of reliable and valid measures that can capture the key components of temperament without placing too high a burden on the time of researchers, professionals, and participants. In line with these desiderata and based on the integrative approach of Zentner and Bates [10], Zentner and Wang [11] developed the *Integrative Child Temperament Inventory* (ICTI), which is available in English and German [12]. The term “integrative” denotes the fact that this measure assesses well-studied child temperament characteristics that are represented across various models of temperament, rather than assessing temperament according to a particular conceptualization of temperament. For an overview of the ICTI dimensions see Table 1. The *Integrative Child Temperament Screener* (ICTS) was recently added as a short form of the ICTI for the measurement of traits that are of particular clinical significance. Indeed, the ICTS has been shown to discriminate between children referred for psychiatric treatment and general population children [13, 14].

**Table 1:** Summary and capsule definitions of temperament dimensions included in the ICTI

ICTI dimensions	Capsule definitions	Related Dimensions
Behavioral inhibition	Inhibition of behavior in response to novel unfamiliar people and situations	Harm avoidance, shyness
Frustration	Aggressive or irritated behavior in response to painful and/or frustrating input	Difficultness, distress to limitations, anger proneness
Activity level	Frequency, speed and vigor of gross motor movement and locomotion; intolerance toward enforced idleness	Briskness, energy
Attention/persistence	Capacity for attentional focusing and control as basis for voluntary behavior including persistence	Effortful control, distractibility, novelty seeking
Sensory sensitivity	Ability to react to sensory stimuli (e.g., visual, auditory or tactile) of low stimulative value; sensitivity to sensory discomfort	Threshold, sensory defensiveness

Table 1 adapted from Zentner and Wang [11]

A certain limitation of both the ICTI and the ICTS is that they can only be used for children up to 8 years of age. Yet, the assessment of temperament in late childhood and early adolescence is at least as important for a number of reasons. First, the prevalence of conduct and behavioral disorders is highest in the early school years (age 6–11 years), while emotional problems (depression/anxiety) are often observed shortly before and during adolescence (age 12–17 years) [15]. Temperament has been found to play a role in several of these disorders. For example, during adolescence, anorexia nervosa in females is associated with higher inhibition and persistence [16], whereas drug and substance use, as well as delinquent behavior, is linked to negative mood, low persistence, and low adaptability [17, 18]. There is also evidence suggesting that difficult temperament traits of boys measured at age 10 to 12 predict aggression and affiliations with delinquent peers at age 14 and drug use at age 16 [19]. Furthermore, it has been found that problematic Internet use (PIU) is related to low effortful control, high anger/frustration, and high shyness in children and adolescents [20].

Clearly, none of these associations are deterministic. Thus, it has been found that the context can buffer the negative effects of these temperament risk-factors. Such corrective effects include family involvement and regularity [21, 22], positive parenting styles that match a child’s given temperamental disposition [23], as well as interventions promoting the child’s self-regulatory skills [6–9]. Therefore, assessing temperament in late childhood may help to identify at-risk children and choose appropriate preventions or interventions.

### Assessing Key Components Of Temperament in Late Childhood and Early Adolescence

In light of the evidence reviewed above, it would be desirable to extend the range of application of the ICTI to late childhood and early adolescence. However, due to children’s rate of development and maturation, the relevance and structure of temperament dimensions cannot be assumed to be the same in early and in late childhood. Indeed, Mervielde and De Pauw [24] found that evidence for the distinctness of certain temperament traits changes between the preschool and the school periods. Therefore, in adapting the ICTI for later childhood, it was important to determine whether the traits assessed by the ICTI (frustration, behavioral inhibition, activity level, attention/persistence, and sensory sensitivity) should also be included in the new instrument, or whether certain dimensions should be added or replaced. On one hand, keeping the type and number of dimensions constant across both instruments has obvious advantages, as it facilitates direct comparisons between results produced by both instruments. This can be particularly important when examining the stability of temperament in longitudinal research or when comparing temperament-to-behavior problem associations in early vs late childhood. On the other hand, adhering to the design of a measure can also compromise its validity and usefulness, if certain temperament dimensions become less distinct or relevant due to developmental processes, or dimensions change in character as children move into late childhood. In devising the new instrument, we attempted to strike a balance between these two types of considerations.

Our reading of the literature suggested that the dimensions assessed in the ICTI remain relevant as children move into later childhood, but that changes to item-content and wording had to be envisaged to make the instrument age-appropriate. One dimension that was not included in the ICTI is positive emotionality. This was because it is often conceptualized as a higher-order factor, whereas the most important component of this factor in early childhood period is activity level. However, Putnam [25] proposed a differentiation of positive emotionality in approach-based positive emotionality (extraversion, surgency, sensation seeking) and non-approach-based positive emotionality (agreeableness, affiliation). Because the former does at least partly overlap with activity level and behavioral inhibition (reversed), we decided to include the non-approach-based affiliation-dimension in the new instrument, rather than both, for reasons of parsimony. Affiliation or sociability, which is characterized by the need for warm and close interpersonal relationships [26], is often part of other questionnaires for this age-group and a potential precursor of agreeableness [27]. Low affiliation in children can predict psychopathology, as it is linked to antisocial behaviors, poor relationship quality and internalizing problems [25].

### The Current Research

In light of the above review and considerations, the aim of the current research was the development and validation of a version of the ICTI that could be used to measure temperament from late childhood to early adolescence. In analogy to the ICTI, we named this adaptation the Integrative Late Childhood Temperament Inventory (ILCTI). To this end, we collected temperament ratings of 8- to 14-year-old children from their German- or English-speaking parents. The internal structure of the questionnaire was investigated with a confirmatory factor analysis. Different forms of reliability were examined including internal consistencies and test–retest reliability. As indicators of convergent validity, we included other temperament and personality scales: the EAS, the SATI, the HSC-Scale, the BFI-KJ. Scales’ relationships to performance at school, and to externalizing and internalizing symptoms (SDQ), served as indicators of criterion validity.

## Methods

### Participants and Procedure

324 German- and 201 English-speaking parents (N = 525; *M*_*age*_ = 41.51 years, *SD*_*age*_ = 7.50) took part in an online survey aimed at collecting ratings of their children’s temperament. Most of the raters were mothers (66%), a lesser proportion fathers (31%), and the remaining 3% were other relatives (e.g., older sister, grandfather, stepmother). Although the intended target age group was 9 to 13 years of age, parents rated children between 8 and 14 years of age (*M* = 10.74, *SD* = 1.67), including 265 boys (51%), 258 girls (49%) and two non-binary children. Separate sample characteristics for German- and English-speaking participants are displayed in Supplementary Table 1. The two samples demonstrated no significant differences in relation to sociodemographic variables. Ratings of personality traits, school performance, and emotional and behavior problems of the child were obtained for 372 children. A subsample of 83 parents took part in a test–retest session that took place 14 to 27 days (*M* = 18.39, *SD* = 3.58) after their initial participation. Participants were recruited via crowdsourcing platforms Prolific and Clickworker and were paid for their participation (65%), whereas the other part (35%) was recruited in schools, sports clubs, via the mail distributer and the summer program for children of the University, and in online forums or Facebook groups for parents.

The ethics committee of the department approved the study and all participants provided informed consent before taking part.

## Materials

### Temperament

*Integrative Late Childhood Temperament Inventory (ILCTI).* Items of the ILCTI were mainly derived from the *Integrative Child Temperament Inventory (ICTI)*, which is available in both German [12] and in English [11]. The ILCTI measures the five ICTI-dimensions (see Table 1) and the new dimension affiliation. The ILCTI items related to the ICTI items in one of three different ways: a reproduction of ICTI with no change; b reformulation of items in an age-appropriate manner; c generation of new items for constructs not included in the ICTI. Revisions of former ICTI items and incorporations for new items were made in a process that included consultation with five schoolteachers and the International Personality Item Pool (IPIP [28]. More detailed information on the initial development of the ILCTI, which also included a pilot study, is included in the Supplementary Materials (SM1). At the end of the development process, the ILCTI included 39 items that are answered by parents or other caretakers on the same six-point answer format of the ICTI ranging from 1 (*behavior occurs never or hardly ever*) to 6 (*behavior occurs always or close to always*).

### Other Temperament Measures

*EAS Temperament Survey for Children.* The parent rating version of the EAS was administered to examine the convergent validity of ILCTI frustration, activity level, affiliation and behavioral inhibition, as it includes four related temperament dimensions: Emotionality, activity, sociability and shyness [29]. The EAS consists of 20 items, five for each dimension, and is rated on a five-point scale from *not characteristic or typical of your child* (1) to *very characteristic or typical of your child* (5).

*School-Age Temperament Inventory (SATI).* The scale “task persistence” of the SATI was included to investigate convergent validity of attention/persistence [30]. It consists of 11 items that are rated from *never* (1) *to always* (5).

*Highly Sensitive Child Scale (HSC Scale).* Convergent validity of sensory sensitivity was examined with the dimension low sensory threshold of the HSC Scale [31]. The nine items are answered on a seven-point scale ranging from 1 (*Not at all*) to 7 (*Very much*).

### Personality

*Big Five Inventory for Children and Adolescents (BFI-K KJ-F).* The other-rating short form of the BFI-K KJ-F measures the Big Five personality traits extraversion, neuroticism, conscientiousness, agreeableness, and openness [32]. 26 items are answered on a five-point scale ranging from *not true at all* (0) to *absolutely true* (4).

### Behavior Problems

*Strengths and Difficulties Questionnaire (SDQ).* The SDQ was used to investigate criterion validity. It assesses the five scales Emotional symptoms, conduct problems, hyperactivity, peer problems and prosocial behavior with 25 items [33]. Each item is rated on a 3-point scale, including *not true* (0), *somewhat true* (1) or *certainly true* (2). Conduct problems and hyperactivity can be summed to generate an externalizing subscale, whereas emotional symptoms and peer problems represent an internalizing subscale. Furthermore, the four scales can be combined into a total difficulties score [34].

### School Performance

To obtain a measure of school performance, we asked the parents the following three questions: (1) “Which grade describes your child’s academic performance best (or which grade did your child receive most often during the last school year)?”; (2) “How do you rate your child’s academic performance compared to his or her classmates? (answer format ranges from 1 (*is below average*) to 4 (*is one of the best in his/her class*))”; and (3) “Does your child find school and learning easy in general?” (answer format ranges from 1 (*No*) to 4 (*Yes*)). Because of the good internal consistency reliability of the three questions (ω = 0.85), the three variables were z-transformed and averaged into a composite called “school performance”.

## Results

### Data Reduction and Internal Structure of the Questionnaire

Prior to the main analyses, two items were omitted due to parents reporting that one item did not suit the target age group (“Stays close to the mother (or another caretaker) when meeting unfamiliar people”), and another item being too unspecific to answer (“Likes to share and to let other children use his/her things.”). The structure underlying the remaining 37 items of the questionnaire was analyzed with confirmatory factor analysis (CFA) in R v4.1.1 (lavaan package), using maximum likelihood estimation. After a preliminary analysis, seven items were discarded: four due to excessive cross-loadings and three further items because they did not load sufficiently on the intended factor.^1^ For a complete overview of the remaining 30 items, their means, standard deviations, and sources see Appendix. The German items are available on request.

To ensure that the number of factors we defined represented the data well, we ran a parallel analysis (PA) in R, a simulation-based method that takes sampling error into account [35]. The PA suggested six factors as anticipated. Subsequently, we ran a CFA in which each factor was represented by 5 items. Factors were allowed to correlate based on previous findings that suggested small to moderate intercorrelations between the six dimensions [11, 12]. Subsequently, we tested the model fit based on (a) the comparative fit index (CFI), (b) the standardized root mean squared residual (SRMR), (c) the root mean square error of approximation (RMSEA), and (d) the chi-square statistic (χ^2^), because these four indicators provide different types of information and are the most commonly used criteria for evaluating fit [36]. According to Kline [37], the fit is acceptable when CFI is ≥ 0.90, SRMR ≤ 0.08, RMSEA ≤ 0.06.

**Fig. 1:**
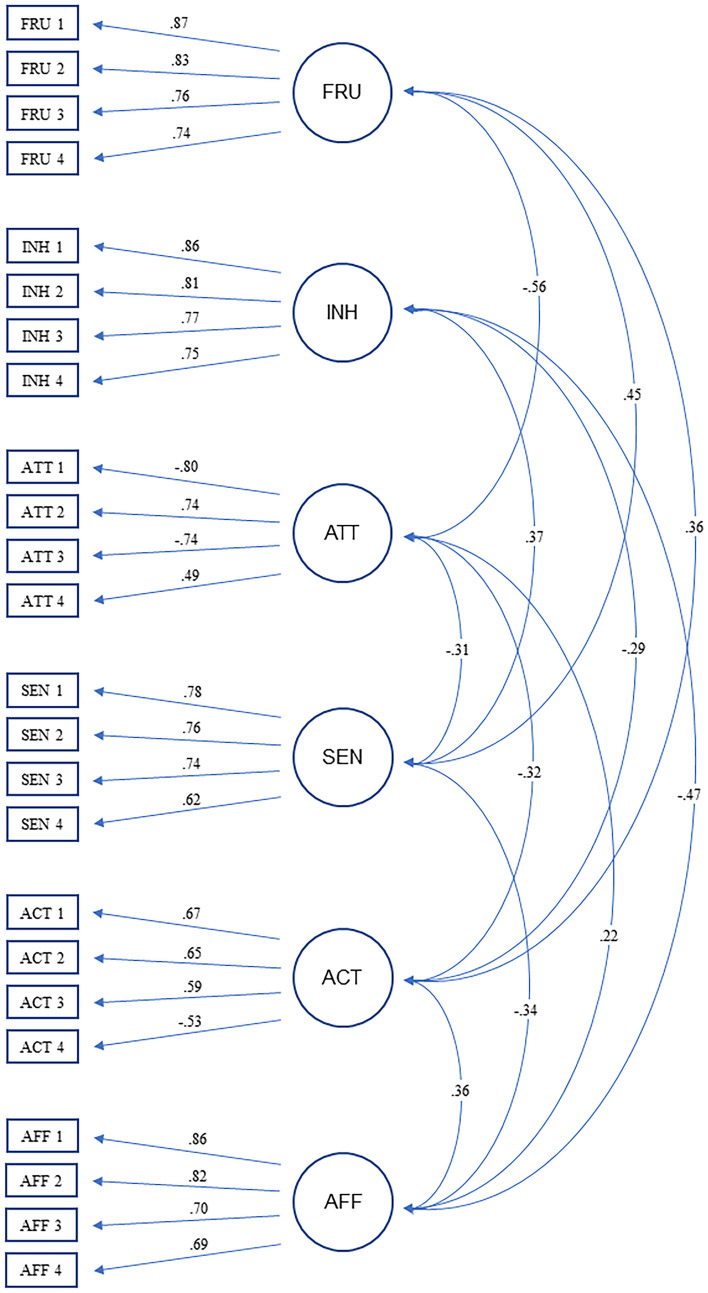
Results of the final measurement model for the 24-item version. Values represent standardized covariances and factor loadings. Correlations beneath .20 are not reported. FRU Anger/Frustration, INH Behavioral Inhibition, ATT Attention/Persistence, SEN Sensory Sensitivity, ACT Activity Level, AFF Affiliation

As can be seen from Table 2, the model fell short of these criteria (CFI = 0.840, SRMR = 0.085, RMSEA = 0.071, χ^2^ (390) = 1418.33, *p* < 0.001). Due to the complexity of personality models, this is not an unusual finding [38]. Still, in an attempt to improve the fit, we removed the item with the poorest performance in each scale (e.g., lowest item-to-total correlation, cross-loadings). The fit indices for the resulting 24-item model were in or close to the recommended range (CFI = 0.915, SRMR = 0.064, RMSEA = 0.057, χ^2^ (237) = 644.00, *p* < 0.001). The significance of the chi-square test does not detract from the acceptable model fit, since even models with small discrepancies tend to yield a significant value [36]. The 24-item version fitted the data significantly better than did the model with 30 items (Δχ^2^ (153) = 774.33, *p* < 0.001). As a consequence, we retained the 24-item version of the questionnaire for additional psychometric scrutiny (see Fig. 1 for the standardized path coefficients).

**Table 2:** Model Fit Indices of the Confirmatory Factor Models for different numbers of ILCTI items

ILCTI Model	CFI	SRMR	RMSEA	*χ*2 (*df*)	Δ*χ*2 (*df*)
30 items (5 items per scale)	.840	.085	.071	1418.33 (390)*	
24 items (4 items per scale)	.915	.064	.057	644.00 (237)*	774.33 (153)*

*Note. N* = 525; *ILCTI* integrative late child temperament inventory, *CFI* comparative fit index, *SRMR* standardized root mean square residual, *RMSEA* root mean square error of approximation, Δ = increment of change

^*^p < .001

### Internal Consistency and Test–Retest Reliability

Next, we assessed the internal consistency and test–retest reliability of the 4-item scales. For internal consistency, we used McDonald’s ω since it is less influenced by commingling populations and makes more realistic assumptions compared to Cronbach’s α [39]. As can be seen in Table 3, all psychometric indicators were slightly inferior to those of the 30-item version, but still acceptable and comparable to the values of the ICTI and ICTS, which internal consistencies ranged between 0.65 and 0.89 and test–retest reliability between 0.67 and 0.91 [11–13].

**Table 3:** Internal consistency reliability and test–retest reliability (Spearman correlation) of the ILCTI Scales for different numbers of items

	Mc Donald’s ω^a^		Test-Retest^b^	
ILCTI Scales	30 items	24 items	30 items	24 items
Frustration	.88	.89	.83*	.81*
Behavioral Inhibition	.88	.86	.77*	.75*
Attention/Persistence	.84	.77	.82*	.80*
Sensory Sensitivity	.84	.82	.79*	.78*
Activity Level	.75	.72	.78*	.72*
Affiliation	.87	.84	.80*	.75*

*Note ILCTI* integrative late child temperament inventory, ^a^*N* = 525; ^b^*N* = 83

^*^p < .001

In light of the psychometric adequacy, we retained the shorter version as the final ILCTI version. Means, standard deviations and intercorrelations of the final ILCTI scales, as well as their correlations with sex and age are reported in Supplementary Table 2.

### Construct and Criterion Validity

Overall, the pattern of convergent and discriminant correlations supported the instrument’s construct validity. As can be seen from Table 4, the ILCTI scales correlated highly with corresponding scales from other temperament questionnaires (*r* = 0.70 to *r* = 0.81), and from the Big Five personality scales (*r* = 0.39 to *r* = 0.82), but only slightly with conceptually unrelated scales.

**Table 4:** Correlations of the ILCTI Scales with the EAS, SATI, HSC Scale and BFI Scales

	ILCTI Frustration	ILCTI Behavioral Inhibition	ILCTI Activity Level	ILCTI Affiliation	ILCTI Attention/Persistence	ILCTI Sensory Sensitivity
EAS Emotionality	.70*	.14	.15	− .18*	− .40*	.45*
EAS Shyness	.06	.73*	− .36*	− .74*	− .08	.29*
EAS Activity	.13	− .39*	.75*	.44*	− .07	− .15
EAS Sociability	.07	− .44*	.36*	.76*	− .05	− .20*
SATI Task Persistence	− .54*	− .09	− .28*	.24*	.81*	− .26*
HSC Scale Low Sensory Threshold	.34*	.41*	.04	-.42*	− .22*	.80*
BFI Extraversion	.02	− .78*	.39*	.53*	− .06	− .28*
BFI Neuroticism	.68*	.34*	.03	− .37*	− .45*	.47*
BFI Conscientiousness	− .52*	− .10	− .14	.20*	.77*	− .23*
BFI Agreeableness	− .14	− .46*	.25*	.82*	.13	− .24*
BFI Openness to Experience	− .12	− .17	.15	.38*	.40*	.08

*Note N* = 372; *ILCTI* integrative late child temperament inventory; *EAS* EAS temperament survey for children; *SATI* school-age temperament inventory; *HSC Scale* highly sensitive child scale; *BFI* big five inventory for children and adolescents

^*^p < *.001*

Importantly, the associations with the clinically relevant SDQ scales (Table 5) conformed with expectations and were also consistent with ICTI-to-SDQ correlations. The only exception was the smaller contribution of behavioral inhibition to emotional problems and therefore internalizing problems. Instead, lack of affiliation accounted for most of the variance of internalizing problems, mainly through its subscale peer problems. Of particular note was the strong association of attention/persistence with school performance, which serves as a reminder of the importance of temperament for success or failure in school. Attention/persistence also turned out to be the strongest predictor for externalizing problems.

**Table 5:** Multiple regression. Unique contributions (standardized beta weights) of ILCTI scales to school performance and the SDQ symptom scales, with child age and gender controlled for

	School Perfor-mance	SDQ Prosocial Behavior	SDQ Conduct Problems	SDQ Hyper-activity	SDQ Emotional Symptoms	SDQ Peer Problems	SDQ Externalizing Problems	SDQ Internalizing Problems
ILCTI Frustration	− .09	− .28*	.63*	.12	.22*	.12	.38*	.21*
ILCTI Behavioral Inhibition	− .05	.03	− .19*	− .04	.22*	− .05	− .11	.12
ILCTI Attention/ Persistence	.47*	.18*	− .16*	− .63*	− .17*	− .07	− .48*	− .14
ILCTI Activity Level	− .07	.07	.07	.29*	− .15	− .02	.23*	− .08
ILCTI Affiliation	.10	.38*	− .13	− .08	− .15	− .66*	− .12	− .44*
ILCTI Sensory Sensitivity	.10	.11	− .05	− .01	.15	.10	-.03	.15*

*Note N* = 372; *ILCTI* integrative late child temperament inventory; *SDQ* strength and difficulties questionnaire. Coefficients are standardized beta weights, representing unique contributions of each temperament dimension to problem scores, with child age and gender controlled for

^*^p < *.001*

## Discussion

The results of this study demonstrate that the ILCTI provides a brief, reliable and valid tool for the assessment of temperament in 8- to 14-year-old children. As such, it offers an important extension of the ICTI, which assesses the same temperament dimensions except of affiliation in 2- to 8-year-old children. Notably, the ILCTI’s internal structure was well supported by CFA, reliability indicators including internal consistencies and test–retest reliability were in the range of those reported for the ICTI and ICTS [11–13]. There were no substantial differences in the means of the dimensions between boys and girls or between English- and German-speaking parents. Convergent and discriminant validity (with other temperament and personality scales) as well criterion validity with the SDQ problem scales and school performance were supported for all six ILCTI dimensions. Importantly, the pattern between the ILCTI scales and the SDQ symptom scales was in line with expectations and previous research [2, 13]. One exception was the comparatively small association between behavioral inhibition and SDQ emotional problems, in contrast to moderate to substantial relations reported in the literature [13], but consistent with studies in late childhood and early adolescence populations, which also found relatively small associations between behavioral inhibition or shyness and emotional symptoms [40, 41]. The addition of the dimension affiliation proved important in light of its substantial relationships with prosocial behavior and peer problems (reversed), which is consistent with the literature on the importance of affiliation for social and school contexts in older children and adolescents [25]. Overall, the magnitude of the associations between temperament traits and externalizing or internalizing symptoms are comparable to those found for the ICTS [13, 14].

### Implications and Use

The ILCTI has advantages for both research and applied settings. First, since it assesses temperament dimensions related to emotional and behavior problems and school failure, it can be helpful in identifying children at risk for these problems. As the ILCTI’s parent instrument, the ICTI, showed good screening accuracy for behavioral problems in terms of sensitivity and specificity [13, 14], there is reason to expect that the ILCTI could be useful as screening tool as well, enriching currently available tools for identifying children at risk in the preadolescent period. This is particularly important given that several disorders (e.g., depression, anxiety) have their onset shortly after in adolescence [15]. Prevention programs for depression in school-aged children are already available [42] and the ILCTI might be helpful in identifying children in need of such prevention. Regarding poor school performance, our findings are in line with those of previous studies, which found that regulative aspects of temperament (e.g., attention, persistence, self-discipline) are often better predictors of school performance than IQ [43]. Because school failure is not only an undesirable outcome in itself but has also been found to be a risk factor for other negative outcomes, such as child and adolescent delinquency [44], identifying these temperament risk factors could be helpful in the context of delinquency prevention.

Second, the ILCTI is a short measure that can be completed in less than 5 min. As such, it can easily be included in larger-scale studies that need to assess temperament as a secondary or control variable. Third, in longitudinal studies it is desirable to assess constructs with commensurate scales throughout the study period. The combined use of the ICTI and ILCTI makes it possible to assess equivalent temperament characteristics from ages 2 to 14 years. Fourth, in children that have been referred for treatment, information on temperament may be useful in selecting an effective treatment method. For example, patients with high levels of traits related to neuroticism and low levels of traits related to agreeableness have been found to respond better to antidepressant medication than to cognitive-behavioral therapy [45]. Although evidence for this type of personalized treatment in children is scarce, one study found that girls at risk of depression with high sensory processing sensitivity responded better to a school-based prevention program than those with low sensory processing sensitivity [46]. Finally, there is now an increasing number of temperament-based interventions that allow temperament-related problems to be matched with temperament-related treatment approaches [6–9], including those that aim to improve children’s self-regulation, which have been found to reduce substance abuse and school failure [47].

### Strengths and Limitations

The results of the present study should be interpreted within certain limitations. First, the collected data was cross-sectional, precluding strong conclusions about the predictive and causal role of the ILCTI temperament dimensions in affecting school performance and behavior problems. Second, the 24-item version of the ILCTI was not administered as a stand-alone questionnaire. However, since there were no correlated residuals in the data, the probability of item order-effects is relatively low. Third, although comparatively ample evidence for the questionnaire’s convergent, discriminant, and criterion validity was obtained in the current study, the validation of any measure is a continuous process that will require different types of independent studies to produce definite results. For example, because parents rated both the child’s temperament and his or her symptoms, the two measures are not strictly independent. One way to address this limitation in future work is to examine the temperament of children that have been referred for psychiatric treatment and/or to include ratings by teachers and the children themselves. Indeed, children from about age nine can provide valid self-ratings on personality and temperament questionnaires [48–50]. We take this as an impetus for deriving and examining a self-report version of the ILCTI in future research. Despite these limitatios, the ILCTI fills a gap in measures of child temperament by providing a brief, reliable, and valid instrument for the assessment of temperament during the late childhood to early adolescent period.

## Summary

Temperament has been found to play a role in several of psychological disorders emerging between late childhood and early adolescence. However, few comprehensive and time-efficient temperament measures exist for this age period. To close this gap, the aim of the current study was to develop a measure capable of assessing six basic dimensions of temperament during this developmental stage. As a point of departure, we used the Integrative Child Temperament Inventory (ICTI) and adapted it for the late childhood and early adolescent period. Rather than reflecting a particular model of temperament, the ICTI provides an integrative measure of temperament components that are found across different child temperament models. The current late childhood version—called Integrative Late Child Temperament Inventory (ILCTI)—was examined on the basis of temperament ratings of 8- to 14-year-old children in large samples of German- or English-speaking parents. Results indicated that, despite comprising 24 items only, the questionnaire showed good psychometric properties: factorial validity, as evidenced by satisfactory CFA model fit, good internal consistency, and good test–retest reliability. Furthermore, we found evidence for the ILCTI’s convergent validity, criterion validity, and clinical utility, with several dimensions showing significant associations with emotional and behavior problems as well as poor school performance. In research settings, the ILCTI meets the demand for a brief yet comprehensive measure of temperament for the late childhood and puberty periods. In applied settings, the ILCTI may be helpful in identifying children at risk, thereby facilitating the application of existing prevention and intervention programs that focus on child temperament and related dispositions. Taken together, the new scale contributes to fill a gap in current measurement tools for identifying behavioral and emotional risk factors in the period stretching from late childhood to early adolescence.

## Electronic supplementary material

Below is the link to the electronic supplementary material.

Supplementary file1 (DOCX 26 KB)

## Data Availability

Data and materials can be requested from the authors.

## Appendix

See Table 6.^2^

**Table 6:** Descriptive statistics and sources of the Integrative Late Childhood Temperament Inventory Items

ILCTI Items	Abbreviation	M	SD	Source of the item
20. Gets angry when he/she is not allowed to do something	FRU1	3.28	1.47	c
14. Gets very loud when upset (e.g., yelling or slamming doors)	FRU2	3.23	1.66	c
8. —^*^	FRU3	3.42	1.40	a
26. —^*^	FRU4	3.51	1.45	a
*2. Is very upset after disappointments or failures*	FRU5	3.61	1.35	c
6. Is shy when meeting unfamiliar children	INH1	3.35	1.50	a
30. Is shy when meeting unfamiliar adults (e.g., at the doctor's office, at sports, friends of parents)	INH2	3.52	1.53	c
12. Does not like to talk in front of many people	INH3	3.47	1.60	b
24. Takes time to warm up to new situations	INH4	3.94	1.24	b
*18. Approaches unknown children spontaneously and joins in their games/activities. (R)*	INH5	3.70	1.46	b
23. Is easily distracted from his/her projects/tasks (e.g., homework). (R)	ATT1	3.37	1.45	b
5. Concentrates on something for long periods without difficulty	ATT2	4.29	1.34	a
11. Starts activities that he/she does not finish (e.g., building or crafting, puzzles). (R)	ATT3	4.10	1.33	c
29. —^*^	ATT4	3.62	1.26	a
*17. Practices new skills until they are mastered (e.g., challenging games, learning sports or playing a new piece of music)*	ATT5	3.82	1.30	b
16. —^*^	SEN1	2.89	1.53	a
10. Is bothered by very bright or glaring light	SEN2	2.49	1.43	c
28. Reacts sensitively to the feel (texture) of clothes (e.g., rough or itchy fabrics)	SEN3	3.10	1.67	b
22. Reacts sensitively to small changes in smell and/or taste (e.g., new recipes, products from different brands.)	SEN4	3.44	1.52	b
*4. Reacts sensitively to the temperature of food/drinks*	SEN5	2.63	1.46	b
25. —^*^	ACT1	3.77	1.27	a
19. Likes to run	ACT2	3.05	1.58	b
13. Is very energetic	ACT3	4.28	1.29	c
7. Likes quiet activities/games better than active ones. (R)	ACT4	3.09	1.32	c
*1. Likes to be physically active and do sports in his/her free time*	ACT5	4.02	1.42	c
27. Likes to be around other children	AFF1	4.79	1.23	c
15. Is something of a loner. (R)	AFF2	4.46	1.46	c
9. Enjoys working with others (e.g., group work at school, team sports)	AFF3	4.30	1.23	c
3. Wants to meet with friends frequently	AFF4	4.12	1.36	c
*21. Close relationships and friendships are very important to him/her*	AFF5	4.63	1.18	c

* Copyrighted items are blanked out

*Note ILCTI* integrative late childhood inventory; items in italic are not retained in the final ILCTI version;* R* reversed item, *Fru* frustration, *Inh* behavioral inhibition, *Att* attention/persistence, *Sen* sensory sensitivity, *Act* activity level, *Aff* affiliation*, **a* original ICTI item; *b* adapted ICTI item; *c* new item from Teachers and/or IPIP

## Footnotes

1 The seven excluded items were the following: “Is even tempered, easy to manage.”, “Stops playing with a difficult game or toy rapidly.”, “Does not like to sit still for long periods (e.g., in the doctor’s waiting room, on long car rides).”, and “Likes trips and hikes.”), “Is bothered when his/her hands are dirty or sticky.”, “Likes to share concerns and thoughts with others.”, and “Notices when others need help.”).
