# Supplementary material for: Assessing Temperament Risk Factors in Late Childhood and Early Adolescence: Development and Validation of the Integrative Late Childhood Temperament Inventory
Source: Child Psychiatry Hum Dev. 2024 Apr 15;57(1):152–62. doi: 10.1007/s10578-024-01675-5 (PMC12971908; doi:10.1007/s10578-024-01675-5)
Supplement: Supplementary file 1 — Supplementary file1 (DOCX 26 KB) [file 10578_2024_1675_MOESM1_ESM.docx]

**Assessing Temperament Risk Factors in Late Childhood and Early Adolescence:**

**Development and Validation of the Integrative Late Childhood Temperament Inventory**

In *Child Psychiatry & Human Development*

Vivienne Biedermann^1^ and Marcel Zentner^1^

^1^Department of Psychology, University of Innsbruck, Innsbruck, Austria

**Correspondence**

Vivienne Biedermann, MSc, Department of Psychology, University of Innsbruck, Universitätsstrasse 15, 6020 Innsbruck, Austria Tel. +43 (0) 512 507-56039

E-mail: [Vivienne.Biedermann@uibk.ac.at](mailto:Vivienne.Biedermann@uibk.ac.at)

ORCID: 0000-0003-4406-6143

**Supplementary Material**

Table S1: *Descriptive statistics of German and English-speaking participants*

|  | German-speaking | | | English-speaking | | | *p* |
| --- | --- | --- | --- | --- | --- | --- | --- |
|  | *N=324* | | | *N=201* | | |  |
|  | | |  | |  | |  |
| Sex | |  | |  | | .500 | |
| Male | | 161 (49.7%) | | 104 (51.7%) | |  | |
| Female | | 161 (49.7%) | | 97 (48.3%) | |  | |
| Non-binary | | 2 (0.6%) | | 0 (0.00%) | |  | |
|  | |  | |  | |  | |
| Age  (at time of first assessment) | | 10.67 (1.69) | | 10.87 (1.62) | | .173 | |
|  | |  | |  | |  | |
| Country of residence | |  | |  | |  | |
| Germany | | 176 (54.3%) | | 0 (0.00%) | |  | |
| Austria | | 143 (44.1%) | | 0 (0.00%) | |  | |
| Switzerland | | 3 (0.9%) | | 0 (0.00%) | |  | |
| Italy | | 2 (0.6%) | | 0 (0.00%) | |  | |
| United Kingdom | | 0 (0.00%) | | 162 (80.6%) | |  | |
| United States | | 0 (0.00%) | | 23 (11.4%) | |  | |
| Canada | | 0 (0.00%) | | 7 (3.5%) | |  | |
| Ireland | | 0 (0.00%) | | 6 (3.0%) | |  | |
| Australia | | 0 (0.00%) | | 3 (1.5%) | |  | |
|  | |  | |  | |  | |

**SM1. Development of the ILCTI**

The ILCTI items related to the ICTI items in one of three different ways: a) reproduction of ICTI with no change; b) reformulation of items in an age-appropriate manner; c) generation of new items for constructs not included in the ICTI. Revisions of former ICTI items and suggestions for new items were made in a process that included consultation with five schoolteachers and an item-database, the International Personality Item Pool (IPIP) [16]. This process resulted in a preliminary version of the inventory, consisting of 37 items, which were administered to a pilot sample of 91 German-speaking parents. They rated their 8- to 13-year-old children (37 girls; M_age_ = 10.15, SD_age_ = 1.39) using the same six-point answer format of the ICTI (more detailed instructions are reported below). The analyses identified a total of 15 items that were either not well-understood by some of the parents (5 items) or did not load unambiguously on their intended factors (10 items). Six of these items were discarded, nine revised, and eight new items added leading to 39 final items (see S1B). For frustration, behavioral inhibition, attention/persistence, and sensory sensitivity, six items were added for each scale. Activity level included seven, and affiliation eight items, as these scales needed more revisions after the pilot testing. Revisions and additions were again made in consultation with teachers and by drawing from relevant IPIP scales. After that, the new German items that were not originally part of the ICTI were translated into English by a bilingual native speaker and then back-translated by another. Discrepancies between the German items and the back-translation were discussed and resolved by both bilingual speakers.

**Instructions**

Please answer the following questions spontaneously, without thinking for too long. There are no good or bad answers. For each question, please circle the answer option that corresponds best to the child’s behavior, as follows:

**1** It never or almost never applies to the child

**2** It seldom applies to the child

**3** It sometimes applies to the child

**4** It fairly frequently applies to the child

**5** It frequently applies to the child

**6** It always or almost always applies to the child

Table S2: *Means, standard deviations, Pearson zero-order correlations, and internal consistencies.*

|  | Variable | M | SD | 1 | 2 | 3 | 4 | 5 | 6 | 7 | 8 |
| --- | --- | --- | --- | --- | --- | --- | --- | --- | --- | --- | --- |
| 1 | Sex (0=boys,  1=girls) | .49 | .50 | - |  |  |  |  |  |  |  |
| 2 | Age (years) | 10.74 | 1.66 | .08 | - |  |  |  |  |  |  |
| 3 | Frustration | 3.36 | 1.25 | -.06 | .00 | (.89) |  |  |  |  |  |
| 4 | Behavioral Inhibition | 3.57 | 1.23 | .01 | .11 | .12 | (.86) |  |  |  |  |
| 5 | Attention/ Persistence | 3.87 | 1.05 | .16* | -.01 | -.41* | -.09 | (.77) |  |  |  |
| 6 | Sensory Sensitivity | 2.98 | 1.19 | -.08 | .04 | .38* | .30* | -.20* | (.82) |  |  |
| 7 | Activity Level | 3.55 | .99 | -.11 | -.15* | .25* | -.22* | -.15* | .08 | (.72) |  |
| 8 | Affiliation | 4.42 | 1.07 | .08 | -.16* | -.09 | -.41* | .19* | -.26* | .27* | (.84) |

*Note. N* = 525; matrix diagonal (in parentheses): McDonald’s omega; *M* = mean; *SD* = standard deviation.

**p* <.001.
